# Dormant non-culturable *Mycobacterium tuberculosis* retains stable low-abundant mRNA

**DOI:** 10.1186/s12864-015-2197-6

**Published:** 2015-11-16

**Authors:** Dmitriy V. Ignatov, Elena G. Salina, Mikhail V. Fursov, Timofey A. Skvortsov, Tatyana L. Azhikina, Arseny S. Kaprelyants

**Affiliations:** Shemyakin and Ovchinnikov Institute of Bioorganic Chemistry, Russian Academy of Sciences, 117997, Miklukho-Maklaya 16/10, GSP-7, Moscow, Russian Federation; A.N. Bakh Institute of Biochemistry, Russian Academy of Science, 119071, Leninsky prospekt 33, Build. 2, Moscow, Russian Federation; Current address: The Queen’s University of Belfast, School of Biological Sciences, Medical Biology Centre, 97 Lisburn Road, Belfast, BT9 7BL UK

**Keywords:** *Mycobacterium tuberculosis*, Dormancy, Non-culturable cells, RNA-seq, Transcriptome

## Abstract

**Background:**

Dormant *Mycobacterium tuberculosis* bacilli are believed to play an important role in latent tuberculosis infection. Previously, we have demonstrated that cultivation of *M. tuberculosis* in K^+^-deficient medium resulted in generation of dormant cells. These bacilli were non-culturable on solid media (a key feature of dormant *M. tuberculosis in vivo*) and characterized by low metabolism and tolerance to anti-tuberculosis drugs. The dormant bacteria demonstrated a high potential to reactivation after K^+^ reintroduction even after prolonged persistence under rifampicin. In this work, we studied the transcriptome and stability of transcripts in persisting dormant bacilli under arrest of mRNA *de novo* synthesis.

**Results:**

RNA-seq-based analysis of the dormant non-culturable population obtained under rifampicin exposure revealed a 30–50-fold decrease of the total mRNA level, indicating global transcriptional repression. However, the analysis of persisting transcripts displayed a cohort of mRNA molecules coding for biosynthetic enzymes, proteins involved in adaptation and repair processes, detoxification, and control of transcription initiation. This ‘dormant transcriptome’ demonstrated considerable stability during *M. tuberculosis* persistence and mRNA *de novo* synthesis arrest. On the contrary, several small non-coding RNAs showed increased abundance on dormancy. Interestingly, *M. tuberculosis* entry into dormancy was accompanied by the cleavage of 23S ribosomal RNA at a specific point located outside the ribosome catalytic center.

**Conclusions:**

Dormant non-culturable *M. tuberculosis* bacilli are characterized by a global transcriptional repression. At the same time, the dormant bacilli retain low-abundant mRNAs, which are considerably stable during in vitro persistence, reflecting their readiness for translation upon early resuscitation steps. Increased abundance of non-coding RNAs on dormancy may indicate their role in the entry into and maintenance of *M. tuberculosis* dormant non-culturable state.

**Electronic supplementary material:**

The online version of this article (doi:10.1186/s12864-015-2197-6) contains supplementary material, which is available to authorized users.

## Background

Despite many years of thorough investigations, the molecular basis of latent tuberculosis (LTB) infection remains elusive. *M. tuberculosis* subpopulation associated with latent infection in humans is believed to be in a dormant state characterized by low metabolic status and transient inability to grow and proliferate [1–4]. The modeling of this specific physiological state has been attempted over years both *in vivo* [5–9] and *in vitro* [10–18]. However, *in vitro* models had limited success, because dormant *M. tuberculosis* bacilli obtained from the majority of these models were fully culturable, whereas mycobacteria isolated from *in vivo* models of LTB infection were in non-culturable (NC) state [3, 19].

We have recently identified K^+^ deficiency as a triggering factor to generate dormant *M. tuberculosis* bacilli that were NC on solid medium. This *M. tuberculosis* population was morphologically distinct, and exhibited tolerance to cell wall-targeting antimicrobial agents. These cells were able to reverse quickly to metabolically active state after K^+^ re-introduction [20]. K^+^ deficiency is a severe stress factor, because K^+^ balance is crucial for many cellular functions, including maintenance of intracellular pH, osmotic pressure, electrochemical gradient, proton-motive force, and activity of the Na^+^/K^+^-transporting ATPase in both eukaryotes and prokaryotes [21–23]. Consequently, the changes in K^+^ availability may induce bacterial responses leading to dormancy and non-culturability, which facilitate long-term survival of *M. tuberculosis in vivo* [20].

Transcriptome profiling of NC mycobacteria by microarray technique revealed downregulation of genes associated with central metabolic processes (e.g., switch of C-metabolism to the glyoxylate shunt, repression of proton-pumping type I NADH dehydrogenase and enzymes of the electron transport chain, and inhibition of ATP synthesis). On the contrary, the genes coding for the uncoupled non-proton-pumping NADH dehydrogenase II (*ndh*) and nitrate reductases (*narG, narH*) were significantly induced in NC *M. tuberculosis*, suggesting that K^+^ limitation prevents mycobacteria from effectively using proton-motive force generated by respiration, thus driving them to utilize NADH dehydrogenase II and alternative electron acceptors. Moreover, a number of genes implicated in amino acid catabolism were upregulated, and the biosynthetic pathways for fatty acids and mycolic acids were downregulated. This suggests a switch to primary metabolites (including amino acids) as a main carbon source, and indicates unique metabolic adaptation of *M. tuberculosis* to the NC state [20].

A general problem for the modeling of *M. tuberculosis* dormancy *in vitro* is the heterogeneity of the bacterial population, which contains cells of different physiological status [24]. Therefore, the elimination of metabolically active cells is a prerequisite for establishing a model of *M. tuberculosis* dormancy. To remove a subpopulation of metabolically active cells (1 × 10^3^ cells ml^−1^) remaining in the population of dormant mycobacteria generated by K^+^ deficiency and to obtain a homogeneous population of NC cells, a 15-day K^+^-starved *M. tuberculosis* culture was treated with a moderate concentration of rifampicin (5 μg ml^−1^), which eliminated dividing cells [25], but did not affect the viability of dormant mycobacteria [15]. As a result, a so-called ‘zero-CFU’ (‘zero - colony forming units’) population of NC *M. tuberculosis* was obtained. The ‘zero-CFU’ population was completely unable to grow on solid medium but retained full potential to recover to physiologically active state, as evidenced by the Most Probable Number (MPN) assay [25]. This highly homogeneous NC *M. tuberculosis* population is much more suitable for transcriptome profiling. Elucidation of specific metabolic processes involved in maintenance of dormant state and preservation of *M. tuberculosis* viability may improve our understanding of LTB infection in humans.

A recently introduced RNA sequencing (RNA-seq) technology based on next-generation sequencing (NGS) overcomes most limitations characteristic for microarray assays. Unlike microarrays, RNA-seq technology does not require transcript-specific probes and allows detection of non-coding transcripts; moreover, RNA-seq determines gene expression levels as digital values [26]. Thus, RNA-seq is a suitable approach for the reliable analysis of dormant transcriptomes with very low mRNA levels.

In this study, we present, for the first time, RNA-seq analysis of the dormant NC *M. tuberculosis* population obtained under K^+^ deficiency, which is characterized by a ‘zero-CFU’ phenotype and high (up to 1 × 10^7^ cells ml^−1^) recovery potential. We compared transcriptional adaptation of *M. tuberculosis* at different stages of dormancy, including the prolonged ‘zero-CFU’ state. Dormant *M. tuberculosis* transcriptome was found to be enriched in small non-coding regulatory RNA molecules. Despite a negligible level of metabolic activity, dormant mycobacteria of the ‘zero-CFU’ phenotype were found to express certain levels of individual transcripts, which were stable in the NC state for at least several days. Protein-coding transcripts stabilized in dormant NC cells could be utilized during further resuscitation steps.

## Results

### Modeling non-culturability

**Fig. 1:**
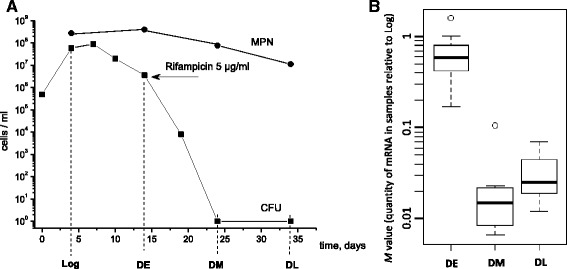
Samples used for RNA-seq analysis. **a** Experimental flow. Formation of a ‘zero-CFU’ *M. tuberculosis* population under K^+^ deficiency and a moderate concentration of rifampicin (5 μg ml^−1^). Filled squares, CFU counts; filled circles, MPN counts. The experiment was repeated five times with similar results. A typical experiment is shown. The standard deviation for CFU did not exceed 10 to 20 % for the CFU mean and 20 to 30 % for the MPN mean. **b** The quantity of total mRNA (*M* values) in the DE, DM, and DL cultures to that in the Log cultures (after normalization to 16S rRNA)

We have recently developed an *in vitro* model of NC *M. tuberculosis* by culturing mycobacteria in K^+^-deficient conditions. After 40 days of incubation, NC mycobacteria were characterized by distinctive morphology and tolerance to cell wall-targeting antibiotics. However these cells were susceptible to rifampicin [20]. The susceptibility of NC mycobacteria to rifampicin may probably be explained by the presence of susceptible subpopulation of active cells remaining in the culture (about 1 × 10^3^–10^4^ cells ml^−1^). Therefore, to enrich NC population and remove dividing cells, rifampicin (5 μg ml^−1^) was added to *M. tuberculosis* cultures K^+^-starved for 14 days [25], which resulted in a gradual decrease in the number of culturable mycobacteria until it reached zero after 10 days of incubation with rifampicin (Fig. 1a). NC cells with the ‘zero-CFU’ phenotype demonstrated high ability to recover metabolic activity and proliferate in the resuscitation medium (up to 2 × 10^7^ cell ml^−1^), as evidenced by the MPN assay, and previously have been successfully used for screening of drugs targeting non-replicating mycobacteria [25].

### The total level of mRNA significantly decreases in dormant cells

Transcriptional profiling of different *M. tuberculosis* populations was performed by RNA-seq. K^+^-deficient cultures at the logarithmic growth phase (Log) were compared with early dormant (DE), and rifampicin-treated ‘zero-CFU’ middle dormant (DM) and late dormant (DL) mycobacteria to determine the changes accompanying K^+^-deficiency, transition to dormancy, and prolonged persistence of dormant NC cells, respectively (Fig. 1a). Three independent replicates allowed estimating biological variability and identifying differentially expressed genes. After sequencing, we mapped the reads to *M. tuberculosis* H37Rv genome, and calculated mapping statistics (Additional file 1: Table S1). The percentages of non-rRNA reads mapped to different annotated features are shown in Table 1. We noted that markedly lower proportion of reads were mapped to protein-coding genes in DM and DL cultures in comparison with DE (*t*-test *p*-values < 0.01 for both comparisons), suggesting a significant decrease of transcriptional activity in ‘zero-CFU’ *M. tuberculosis* cells. Therefore, to obtain unbiased RPKM values, we normalized the number of reads mapped to a protein-coding gene to the number of reads mapped to all protein-coding and non-coding RNA genes, excluding reads mapped to rRNA, and tRNA genes. RPKM values were not calculated for duplicated genes or instances, where less than five reads were mapped to a gene (Additional file 2: Table S2). To compare correlation within and between replicates, we selected the genes with assigned RPKM values for all samples and calculated Spearman correlation coefficients (Additional file 3: Table S3). All samples within triplicates demonstrated high correlation (R > 0.85). Comparison between replicates showed that DM and DL cultures were very similar in their transcriptional profiles (R > 0.85) and might even be considered to represent the same biological sample, indicating that the protein-coding transcriptome of dormant *M. tuberculosis* did not significantly change after prolonged persistence. Interestingly, DE and DM cultures showed slightly higher correlation (R = 0.81) than Log and DE cultures (R = 0.75). The difference between correlation coefficients was statistically significant (Steiger's Z-test, *p* < 0.01) [27]. Therefore, the transition of mycobacteria to early dormancy (when about 90 % *M. tuberculosis* became NC; Fig. 1a) induced more pronounced changes in the transcriptome than the conversion of the remaining 10 % of dividing mycobacteria in population to NC state and formation of ‘zero-CFU’ culture, which is consistent with quantitative assessment of the population structure.

**Table 1:** Mapping statistics

	Log	DE	DM	DL
CDS (% non-rRNA)	80.1	60.2	12.3	18.5
tRNA (% non-rRNA)	3.3	1.7	41.5	13.8
rrnB, 4.5S and tmRNA (% non-rRNA)	14.9	26.3	41.0	61.3
Intergenic RNA (% non-rRNA)	1.6	11.8	5.1	6.4
Intergenic RNA (% sense CDS)	2.0	19.7	41.7	34.7
Antisense RNA (% sense CDS)	13.1	17.7	30.8	35.8

The numbers in the rows one to four represent the percentage of non-rRNA reads mapped to the indicated RNA types for each of the four *M. tuberculosis* states. The percentage of reads mapped to intergenic and antisense RNA to the reads mapped to coding sequences (CDS) in sense orientation are shown in the fifth and sixth rows

Our RNA-seq mapping statistics suggested that the total level of mRNA significantly decreased in DM and DL transcriptomes. The change of mRNA content per cell may result from: (i) the change of the total quantity of RNA per cell, and (ii) the change of proportion of mRNA in the transcriptome. To estimate the first component, we normalized the quantity of RNA isolated from Log and DM cells to the quantity of DNA isolated from these cells. The quantity of DNA proved to be a robust parameter for estimation of the number of cells for mycobacteria [28]. In slow-growing mycobacteria, the quantity of DNA per cell seems to be relatively constant at different growth rates: *M. bovis* BCG was predicted to contain 6.73 fg of DNA per cell at a doubling time 23.1 h^−1^ and 5.35 fg of DNA per cell at a doubling time 69.3 h^−1^ [29]. Therefore, we may assume that in our model the quantity of DNA per cell remained constant. Our results showed that RNA/DNA ratio in Log cells (0.17 ± 0.03, *n* = 3) was almost equal to RNA/DNA ratio in DM cells, cultivated under rifampicin (0.14 ± 0.03, *n* = 3). These data suggested that the quantity of RNA per cell remained constant at different time points in our model.

To estimate the change of proportion of mRNA in the transcriptome we normalized the quantity of total mRNA to the quantity of 16S ribosomal RNA. The total mRNA pool represents a minor proportion (approximately 2 %) of the transcriptome in bacteria [30]. The proportion of mRNA of a certain gene in the transcriptome (*Q*) is composed of (a) the proportion of total mRNA in the transcriptome (*mRNA*) and (b) the proportion of this mRNA molecule in total mRNA pool, which may be represented by RPKM value. Therefore, if we compare samples *A* and *B*, the change of proportion of individual mRNA in total transcriptome (*Q*_*B*_*/Q*_*A*_) may be represented as:

$$ \frac{Q_B}{Q_A} = \frac{mRN{A}_B\times RPK{M}_B}{mRN{A}_A\times RPK{M}_A} $$

To measure the change of proportion of individual mRNA in total transcriptome (*Q*_*B*_*/Q*_*A*_) we employed qRT-PCR with 16S rRNA as a reference gene. Ribosomal RNAs constitute a dominant fraction (more than 80 %) of the total RNA in bacterial cells [30] and rRNA levels are considered to be relatively constant at most growth rates [31]. Therefore, in the condition of decreased mRNA content, 16S rRNA is an appropriate reference gene to calculate the quantity of individual mRNAs in total transcriptome.

To represent the ratio of total mRNA in sample B to that in sample A we introduced the value *M*_*B/A*_:

$$ {M}_{B/A}=\frac{mRN{A}_B}{mRN{A}_A} = qPC{R}_{B/A}^{16S}\times \frac{RPK{M}_A}{RPK{M}_B} $$

We chose 12 genes and measured changes of their expression in samples DE, DM and DL relative to Log by qRT-PCR, using 16S rRNA gene as a reference (Additional file 4: Table S4). As we were unable to obtain PCR products for the genes with low RPKM values in DM and DL samples, highly expressed (overrepresented) genes were chosen for the analysis. Spearman correlation coefficients between changes of expression measured by RPKM and qPCR were: 0.84 (DE vs. Log), 0.87 (DM vs. Log), and 0.88 (DL vs. Log). On the basis of qPCR data and RPKM for 12 selected genes we calculated *M-values* for samples DE, DM and DL relative to Log (Fig. 1b). According to average M-values, the quantity of mRNA relative to 16S rRNA was maximal in Log, slightly decreased in DE, and significantly decreased (30–50-fold) in DM and DL cultures.

### Differential expression of protein-coding genes on dormancy

Despite significant transcriptional repression in DM and DL cultures, the expression of protein-coding genes in these samples could be assessed by RNA-seq. To find differentially expressed genes we used edgeR software [32] (Additional file 5: Table S5). This algorithm does not rely on RPKM. Therefore, there are differences between fold changes calculated with edgeR and by direct comparison of RPKM values. We performed pair-wise comparisons between Log and DE transcriptomes (adaptation to K^+^-deficiency and the early stages of dormancy), DE and DM transcriptomes (formation of the ‘zero-CFU’ phenotype), and DM and DL transcriptomes (prolonged persistence of ‘zero-CFU’ cells).

After calculating differential expression values, we normalized them to the quantity of total mRNA using M-values (Additional file 5: Table S5). We noted, however, that there were no protein-coding genes, whose absolute expression level increased or remained at the same level in DM or DL relative to DE. Additionally, earlier we noted that transcriptional profiles in DM and DL were quite similar to that in DE, suggesting that despite significant downregulation, the relative representation of transcripts in mRNA pool did not show dramatic changes. This prompted us to examine differential expression using non-normalized expression values. In this case, we measured changes of relative representation of individual transcripts in the total mRNA pool, but not the absolute quantities of the transcripts in cells. To facilitate the search for meaningful changes in gene expression, we implemented gene set enrichment analysis using functional categories from TubercuList and PATRIC databases [33, 34]. For this analysis, we chose the genes that showed more than two-fold statistically significant up- or downregulation. The results of functional enrichment analysis are shown in Additional file 6: Table S6.

#### Comparison of Log and DE cultures

**Fig. 2:**
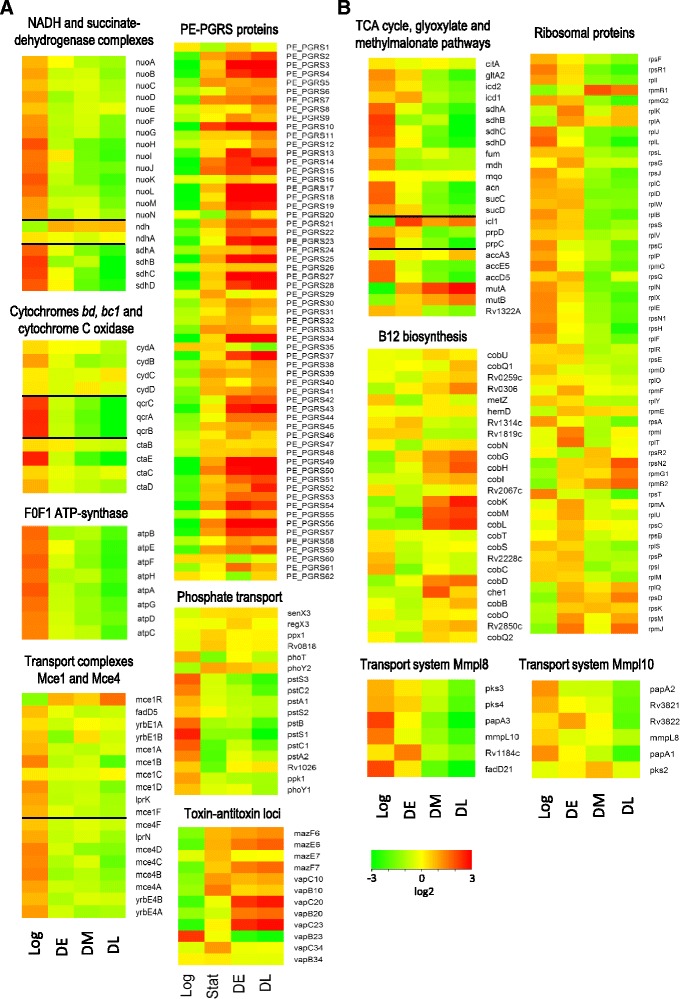
Red-green display summarizing regulation of the selected genes. Gene expression ratios were determined using the edgeR package, log2 transformed, normalized to the average level across the samples and displayed according to the color code. Gene groups were retrieved from the PATRIC database and modified. **a** The groups of genes with differential representation in the Log and DE samples. **b** The groups of genes with differential representation in the DE and DM samples, and DM and DL samples

K^+^-deficient conditions reduced the levels of transcripts encoding NADH dehydrogenase complex I (*nuoA-N*) and succinate dehydrogenase complex (*sdhABCD*), whereas the genes encoding two copies of NADH-dehydrogenase complex II were either significantly upregulated (*ndh*) or unchanged (*ndhA*) (Fig. 2a). We also observed a significant downregulation of expression of the components of the cytochrome *c* pathway in the electron transport chain such as *bc1* complex (*qcrCAB*) and *aa*_*3*_-type cytochrome *c* oxidase (*ctaBCDE*). In contrast, the genes encoding cytochrome *bd* (*cydABCD*) were expressed at the same level. The genes encoding the components of F0F1 ATP-synthase were also downregulated during cultivation in K^+^-deficient media. Other groups of genes that showed significant downregulation were those encoding transport complexes Mce1 and Mce4, and proteins implicated in phosphate transport. *PE-PGRS* genes showed dramatic changes: among 62 genes, 51 showed statistically significant upregulation in DE cells, and most of them remained upregulated in DM and DL cells, where *PE-PGRS* 5, 10, 14, 15, 25, and 41 were expressed at relatively high levels.

#### Comparison of DE and DM cultures

After formation of mycobacteria with ‘zero-CFU’ phenotype (DM cultures), the expression of genes encoding enzymes of the tricarboxylic acid cycle, glyoxylate and methylmalonate pathways was significantly repressed (Fig. 2b). This tendency was already detected in DE cultures and became more pronounced DM and DL. In contrast, *icl1* encoding isocitratelyase and *mutAB* encoding methylmalonyl-CoA mutase, the key enzymes of glyoxylate and methylmalonyl pathways, respectively, were upregulated in DE mycobacteria and remained upregulated in DM and DL cultures. The genes encoding ribosomal proteins and *mmpL8* gene cluster were significantly downregulated in DM and DL cells.

The only group of genes that showed statistically significant upregulation was the group of genes encoding vitamin B12-synthesizing enzymes including cobalamin biosynthetic operon *cobJHKLM*.

#### Comparison of DM and DL cultures

As shown above, both DM and DL mycobacteria were characterized by equally low RNA content and similar transcriptional profiles. Nevertheless, we were able to identify 293 genes with more than two-fold statistically significant up- and downregulation (Additional file 5: Table S5). *mmpL10* gene cluster was significantly downregulated in DL relative to DE (Fig. 2b). Surprisingly, some genes involved in the biosynthetic processes, including *ribD* (riboflavin biosynthesis), *cysO* (cysteine biosynthesis), *grcC2* (polyprenyl diphosphate synthase), and *pckA* (iron-regulated phosphoenolpyruvate carboxykinase, a rate-limiting gluconeogenic enzyme) were upregulated in persisting NC *M. tuberculosis*. The transcripts of several genes involved in lipid and fatty acid metabolism such as *desA2* and *desA3* (desaturases), *fadE23* and *fadE24* (acyl CoA dehydrogenase, lipid degradation), *ech14* (enoyl CoA hydratase), *fabD* (malonyl CoA hydratase), *hsaE* (lipid degradation), and *pks10* (chalcone synthase) were also upregulated during the transition from the middle dormant (DM) to late dormant (DL) state*.*

We also found that several genes involved in the expression and function of ribosomal proteins, including *rpmJ* and *rpmI*, *rpsQ* and *rpsM*, *rplK* and *rbfA* (ribosome-binding factors), *greA* (transcriptional elongation factor), *sigL* and *rsbW* (anti-sigma factors) were upregulated on late dormancy. The genes involved in cell adaptation and repair, including those coding for chaperons, heat shock proteins, and toxin-antitoxin (TA) modules, were also found to be markedly overrepresented on late dormancy: *msrB* (peptide methionine sulfoxide reductase), *serB1* (phosphoserine phosphatase), *hsp* (heat shock protein), *htpX* (peptidase involved in adaptation), *clpB* (ATP-dependent endopeptidase), *pip* (proline release from short peptides), *vapBC10*, and *vapBC20* (TA modules)*.* The genes involved in detoxification reactions such as *cyp132* and *cyp130,* and protein degradation such as *gcvB* (glycine dehydrogenase) and *mpa* (proteasome) also demonstrated upregulation in DL versus DM cells.

### Non-coding transcriptome

Recent studies have identified multiple non-coding RNAs (ncRNAs) in *M. tuberculosis* [35–40]. According to our mapping statistics (Table 1), the quantity of intergenic transcripts (including small RNAs, 5′ and 3′ UTRs) and the relative quantity of antisense RNAs to protein-coding transcripts increased in dormant *M. tuberculosis*, suggesting that ncRNA molecules are upregulated relative to protein-coding transcripts.

By visual inspection of transcriptional profiles, we identified 37 candidate ncRNA molecules, including 8 intergenic small RNAs and 29 antisense RNAs (Additional file 7: Table S7). All intergenic small RNAs and two antisense RNAs have been previously reported [35–38].

According to RPKM values several ncRNAs demonstrated increased expression in dormant cells (Additional file 7: Table S7): ncRv0539c (antisense to putative dolichyl-phosphate sugar synthase) was 10 times upregulated in DE cells; ncRv1162c (antisense to *narH*) was 20 times upregulated in DE cells followed by moderate downregulation in DM and DL cells; ncRv12659 (the transcript originating within a prophage) [41] was 6 times upregulated in DE and further increased in DM and DL cells.

**Fig. 3:**
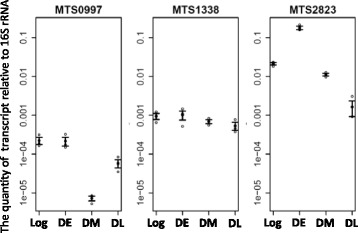
Expression levels of small RNAs MTS0997, MTS1338, and MTS2823. Expression levels were determined by qPCR using 16S rRNA as a reference. Expression levels for three replicates along with the standard error of the mean are shown on the plots

Since small intergenic RNAs MTS0997, MTS1338, and MTS2823 were found to be most abundant in dormant NC mycobacteria (Additional file 7: Table S7), we analyzed their expression at different dormancy stages using qRT-PCR (Fig. 3). Expression of MTS0997 remained constant in DE, but was significantly decreased in DM and DL cells. MTS2823 demonstrated significant accumulation in DE cultures, whereas MTS1338 level remained constant throughout the dormancy, in contrast to the general transcriptional repression in dormant *M. tuberculosis*. Thus, the dormant transcriptome of mycobacteria was proved to be enriched in ncRNAs.

### Overexpression of M. tuberculosis non-coding RNAs affects the growth rate

**Fig. 4:**
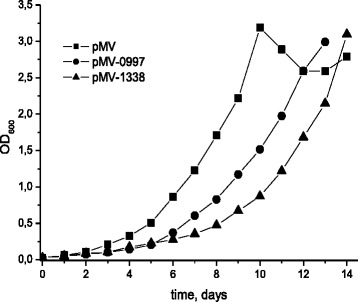
Growth curves of *M. tuberculosis* overexpressing MTS0997 and MTS1338. Mycobacteria were cultured in Sauton medium supplemented with ADC and Tween-80 at 37 °C with agitation (200 rpm); *M. tuberculosis* transformed with the empty pMV261 vector was used as negative control

Next, we examined functional roles of small RNAs in *M. tuberculosis*. Two highly expressed small RNA molecules MTS0997 and MTS1338 were cloned in the plasmid vector under the control of the strong *rrnB* promoter of *M. smegmatis* and transformed into *M. tuberculosis* cells*.* The overexpression of both MTS0997 and MTS1338 in *M. tuberculosis* resulted in slow bacterial growth in liquid medium (Fig. 4) with more pronounced phenotype for MTS1338.

### Cleavage of 23S rRNA is a hallmark of dormant cells

**Fig. 5:**
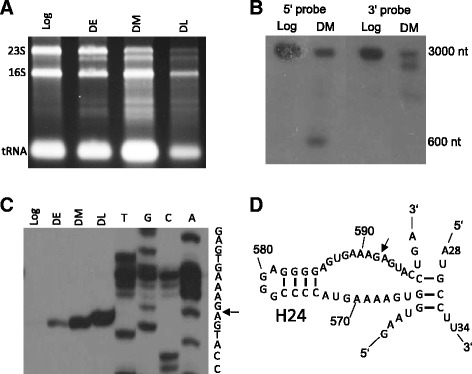
Cleavage of 23S rRNA. **a** Non-denaturing agarose gel electrophoresis of total RNA isolated from the Log, DE, DM, and DL *M. tuberculosis* cultures. **b** Northern blotting with the probes complementary to 5′ and 3′ ends of 23S rRNA from the Log and DM cells. **c** Primer extension analysis with the primer PE_667 complementary to 23S rRNA. RNA was isolated from the Log, DE, DM, and DL cells. The cloned 23S rDNA fragment was also analyzed by Sanger sequencing with PE_667. The arrow indicates the cleavage site. **d** The structure of helix 24 and the adjacent region of *M. tuberculosis* 23S rRNA. The cleavage site is indicated by the arrow. The structure was adopted from www.rna.icmb.utexas.edu [78] and modified

The examination of total RNA quality by gel electrophoresis revealed the fragmentation of 23S rRNA in DE, DM, and DL cultures (Fig. 5a). Northern blot analysis showed that the major cleavage point was located approximately 600 nt from the 5′ end of 23S rRNA. Almost half of the 23S rRNA molecules in DM bacteria were cleaved (Fig. 5b). The primer extension assay was used to map the exact position of the cleavage site between residues G592 and A593. The cleavage occurred in DE cultures, i.e., during the initial phase of dormancy, and became more pronounced in DM and DL mycobacteria (Fig. 5c). In 23S rRNA, the cleavage site is located in a single-strand region near helix 24 of domain I (Fig. 5d); however, this region does not participate in the catalytic activity of ribosomes. Therefore, the role of site-specific 23S rRNA cleavage in *M. tuberculosis* dormancy remains to be elucidated.

## Discussion

To perform transcriptional profiling of dormant NC cells by RNA-seq, we employed a previously developed model of non-culturability in K^+^-deficient medium, which has been earlier used for transcriptome analysis of dormant versus dividing mycobacteria by microarray [20]. However, in the previous study we used a heterogeneous population containing, along with NC cells, a minor subset of dividing cells (1 × 10^3^–10^4^ cells ml^−1^). The addition of a moderate concentration of rifampicin allowed to eliminate this subset of metabolically active mycobacteria [25] and to obtain a homogenous population of dormant cells with the ‘zero-CFU’ phenotype.

In the present study, we demonstrated a significant decrease in total mRNA content in dormant *M. tuberculosis* (especially after establishing ‘zero-CFU’ state in DM and DL cultures), which indicates global repression of all protein-coding genes on dormancy. The problem of comparing gene expression levels in samples with different mRNA content has been recently addressed for eukaryotic cells. Aanes et al. proposed an approach that uses experimentally measured polyA^+^ RNA amounts as scales to normalize expression levels in different samples [42]. Another strategy employed RNA spike-in controls in quantities proportional to the number of cells and estimated scaling factors based on local regression (LOESS) [43].

In this study, we employed a two-step approach: firstly, we calculated RNA to DNA ratio as a measure of total RNA content per cell in compared samples. Secondly, we normalized the quantity of total mRNA to the quantity of 16S ribosomal RNA in compared samples. Our results suggested that the quantity of RNA per cell was equal at different points in our model. However, dormant cells DM and DL with the ‘zero-CFU’ phenotype experienced 30–50 fold downregulation of total mRNA. The downregulation of transcription was so dramatic that there were no genes, whose expression was upregulated in absolute values. Therefore, we performed differential expression analysis in terms of relative representation of particular transcripts in the whole mRNA pool, and not their absolute quantities per cell.

The observed global transcriptional repression of protein-coding genes may be a strategy to maintain dormancy by arresting cell metabolic activity. What is the nature of mRNAs detected in NC cells? It is known that under normal conditions, individual transcripts in bacterial cells have a very short lifetime [44–46], and the presence of mRNA in NC mycobacteria in the absence of RNA synthesis appeared rather unexpected. Hu et al. were the first to demonstrate the preservation of several individual mRNA molecules in rifampicin-treated *M. tuberculosis* cells using RT-PCR; however, global transcriptome was not analyzed [13]. A recent study has shown a significant increase in the average mRNA half-life in *M. tuberculosis* with the decrease in cultivation temperature and under hypoxic conditions [47]. In our model of non-culturable *M. tuberculosis*, we observed a dramatic decrease in transcriptional activity for dormant mycobacteria as indicated by the rate of uracil incorporation [25]. However, we found that dormant NC cells are characterized by the low-abundance but stable transcriptome for at least 10 days of persistence in the ‘zero-CFU’ state. The population of stable individual transcripts in dormant *M. tuberculosis* probably represents mRNA molecules synthesized in the early stages of K^+^-deficiency and stabilized in the NC state, when *de novo* RNA synthesis is evidently blocked.

The analysis of persisting transcripts with significantly increased (≥2-fold) relative representation in the late dormancy revealed a cohort of mRNA molecules coding for biosynthetic enzymes, proteins involved in adaptation and repair processes, detoxification, and control of transcription initiation. Although active metabolic reactions are suppressed in dormant NC *M. tuberculosis* with the ‘zero-CFU’ phenotype, this cohort of stable mRNA molecules could represent a pool of readily activable transcripts preserved for the initial stage of resuscitation as it has been suggested for mRNA found in *Bacillus subtilis* spores [48].

The most prominent feature of the transcriptome from *M. tuberculosis* with ‘zero-CFU’ phenotype was downregulation of the genes encoding ribosomal proteins (Fig. 2b). A similar transcriptional signature has previously been observed during starvation [12], stationary phase, in the Wayne model [49], and in persistent mycobacteria after antibiotic treatment [50]. In our model, the decrease in transcripts encoding ribosomal proteins occurred only after the transition to ‘zero-CFU’ state and not as an early response to К^+^-deficiency, which may represent a specific feature of *M. tuberculosis* adaptation to prolonged dormancy, when only the cells not expressing ribosomal proteins could survive.

A striking feature of the NC transcriptome is a significant proportion of small ncRNAs. Recently, multiple ncRNA molecules were discovered in *M. tuberculosis* [35–37, 39, 51, 52], where they are thought to participate in the regulation of general stress responses [36, 53, 54]. However, the investigation of the roles of small ncRNAs in *M. tuberculosis* has just started [35, 41, 55]. In the present study, two small RNA molecules MTS0997 and MTS1338 were found to be very abundant in NC mycobacteria characterized by the ‘zero-CFU’ phenotype. Both MTS0997 and MTS1338 have been identified earlier in *M. tuberculosis* and *M. bovis* [36, 37, 56]. It has been shown that these small RNAs are induced in the stationary phase of mycobacterial cultures [35], which is consistent with our results demonstrating that MTS0997 and MTS1338 overexpression resulted in growth inhibition (Fig. 4), suggesting the role of these small RNAs in the transition to dormancy and maintenance of the NC status. Arnvig et al. have demonstrated that overexpression of another small RNA, MTS2823, also resulted in slow growth of *M. tuberculosis* [36]. Interestingly, MTS0997 is considered to be involved in cAMP-dependent regulatory processes. Expression of this small RNA is dependent on the synthesis of adenylate cyclase (Rv1264) and cAMP levels [56]. We have recently demonstrated that intracellular cAMP levels regulate the early stages of *M. smegmatis* resuscitation from the dormant NC state [57]. Cumulatively, these data suggest that MTS0997 may be involved in the control of cAMP production in *M. tuberculosis* and thus may participate in the transition to a slow-metabolism state and in the formation of the NC phenotype, when abundant MTS0997 expression would arrest NC cell growth until conditions are appropriate for resuscitation.

Another feature of dormant *M. tuberculosis* was the cleavage of 23S rRNA between residues G592 and A593. Generally, rRNA cleavage by endoribonucleases initiates its degradation during stress conditions, leading to the accumulation of small rRNA fragments for further degradation to mononucleotides by exoribonucleases [58, 59]. If uncontrolled, this process is detrimental and may lead to cell death [60, 61]. During hypoxia, mycobacteria stabilize their ribosomes by keeping 30S and 50S ribosomal subunits in the associated form; the inability to stabilize ribosomes results in their degradation and loss of cell viability [61]. In our model, rRNA remained fragmented but not completely degraded even after prolonged persistence in the NC state. The cleavage site is located in a single-strand region near helix 24 of domain I, which could interact with ribosomal proteins L22 and L24 [62]. The analysis of *E. coli* ribosome crystal structure (PDB ID 2QBE) has shown that the region around the cleavage site is surface-exposed [63] and thus would be readily accessible for proteases.

Ribosomal RNA cleavage may be due to the activity of the toxin-antitoxin (TA) systems of *M. tuberculosis* [11, 64]. Thus, it has been shown that mycobacterial toxin MazF-mt6 can disable protein synthesis by cleaving 23S rRNA at a functionally essential region located in ribosomal A site [65] and that toxin VapC20 can inhibit translation by the cleavage of the Sarcin-Ricin loop in 23S rRNA [66]. In our model, several TA modules such as *mazEF6*, *mazEF7*, *VapBC10*, *VapBC20*, *VapBC23*, and *VapBC34* demonstrated significant upregulation in dormant cells (Fig. 2a).

Despite significant upregulation of *mazEF6* and *vapBC20* TA loci, we did not observe cleavage of 23S rRNA at the cleavage sites specific for these toxins. Indeed, activity of TA systems is mainly regulated at posttranscriptional level: degradation of antitoxin proteins induces activity of toxins [67]. Therefore, transcriptional upregulation does not necessarily indicate activation of TA modules.

## Conclusions

Transcriptomic profiling of dormant NC *M. tuberculosis* by RNA-seq revealed, for the first time, a global transcriptional repression of protein coding genes. The analysis of residual low-abundant transcripts displayed a cohort of mRNA molecules coding for biosynthetic enzymes, proteins involved in adaptation and repair processes, detoxification, and control of transcription initiation. A remarkable transcriptome stability of long-persisting dormant mycobacteria found in this study suggests effective adaptation mechanisms underlying readiness of NC mycobacteria to resuscitation. The mRNAs in dormant cells may represent a pool of stable transcripts, which are rapidly translated upon resuscitation from dormancy. The cleavage of 23S rRNA at a specific point and the abundance of several small ncRNAs in NC *M. tuberculosis* may indicate their significance for the maintenance of dormancy and suggest the molecular basis of LTB infection.

## Methods

### Bacteria and media

Dormant non-culturable *M. tuberculosis* was obtained as described previously [20]. Briefly, *M. tuberculosis* strain H37Rv was initially grown from frozen stocks for 10 days in Sauton medium containing (per liter): 0.5 g KH_2_PO_4_, 1.4 g MgSO_4_ · 7H_2_O, 4 g L-asparagine, 60 ml glycerol, 0.05 g ferric ammonium citrate, 2 g sodium citrate, 0.1 ml 1 % ZnSO_4_ · 7H_2_O, pH 7.0 (adjusted with 1 M NaOH) and supplemented with albumin, glucose and NaCl (ADC) [68] and 0.05 % Tween 80, at 37 °C with agitation (200 rpm). The starter culture was inoculated into fresh medium (same composition) and incubated for another 10 days until its optical density at 600 nm (OD_600_) reached 4.0. These bacteria were then inoculated (5 × 10^5^ cells ml^−1^) into K^+^-deficient Sauton medium (containing 8.9 g Na_2_HPO_4_ · 12 H_2_O instead of 0.5 g KH_2_PO_4_) and grown at 37 °C, 200 rpm. After 14–15 days of culture, when CFU started to decrease, rifampicin (5 μg ml^−1^) was added to eliminate culturable bacteria and to obtain the NC population with the ‘zero-CFU’ phenotype.

### Cell viability estimation

To assess cell viability, 10-fold serial dilutions of *M. tuberculosis* cultures were plated in triplicate onto solidified Sauton agar supplemented with ADC and incubated at 37 °C for 25 days, after which colony-forming units (CFUs) were counted. To assess the proportion of bacteria with the ability to resuscitate in liquid medium by most probable numbers (MPN) assay, 10-fold bacterial dilutions were resuspended in ADC-supplemented Sauton medium diluted 1:1 (v/v; final glycerol concentration, 0.6 %) [18] and seeded into 48-well Corning microplates, which were incubated statically at 37 °C for 30 days. The wells with visible bacterial growth were counted as positive, and MPN values were calculated using standard statistical methods [69].

### Isolation of RNA and DNA, Illumina sequencing

RNA was isolated from *M. tuberculosis* cultures at various time points during initial growth in K^+^-deficient medium and after addition of rifampicin: cells at the logarithmic phase after 4 days in K^+^-deficient medium (Log); early dormant cells at the stationary phase after14 days in K^+^-deficient medium (DE); middle dormant cells (‘zero-CFU’ phenotype) 10 days after the addition of rifampicin (DM); and late dormant cells (‘zero-CFU’ phenotype) 20 days after the addition of rifampicin (DL). Each time point was represented by three independently grown cultures. Bacterial cultures were rapidly cooled on ice, centrifuged, and total RNA was isolated by phenol-chloroform extraction and cell disruption with BeadBeater (BioSpec Products, Bartlesville, OK, USA) as previously described [70]. DNA was isolated from triplicates of Log and DM cultures as described previously [71]. After isolation, RNA was treated with Turbo DNase (Life Technologies, Carlsbad, CA, USA) to remove traces of genomic DNA, and purified with the RNeasy mini kit (Qiagen, Venlo, Netherlands). RNA preparations were depleted of 16S and 23S rRNA using the MicrobExpress kit (Life Technologies) (samples Log 1–3, DM 1–3, and DE 1) or Ribo-Zero (Epicentre, Madison, WI, USA) (samples DE 2, 3 and DL 1–3). cDNA for Illumina sequencing was prepared according to the Directional mRNA-seq Sample Preparation guide (Part # 15018460 Rev. A) as previously described [72]. This procedure preserves strand specificity by ligation of a single-stranded 3′ RNA adapter and 5′ DNA adapter. Sequencing was performed by running 77 cycles on HiSeq 2000 sequencer (Illumina, San Diego, CA, USA).

### Processing of RNA-seq data

The reads were aligned to *M. tuberculosis* reference sequence H37Rv (GenBank accession number AL123456.3) with Bowtie 2 [73] setting parameters *-q –local*; gene annotations were also retrieved from AL123456.3. Reads mapped to several different loci were discarded. Mapping statistics and reads per Kb per million (RPKM) values [74] for annotated genes were calculated using custom Perl scripts. Reads overlapping a gene by at least one nucleotide were counted when calculating gene expression as RPKM. To calculate RPKM values we used “effective” gene length, to which the reads could be unambiguously mapped. To ascertain ‘effective‘gene length, 77 nucleotide sequences were simulated from the genome sequence and aligned to the sequence under the settings used for mapping RNA-seq reads. RPKM values were not calculated for duplicated genes and for genes to which less than 5 reads were mapped. Transcriptional profiles for the forward and reverse strands of the genome representing the counts of overlapping reads for every nucleotide were generated. Transcriptional profiles were visualized with the Artemis genome browser [75]. The differential expression analysis was conducted using the edgeR package [32], and distribution of *M. tuberculosis* genes to functional categories was performed based on the TubercuList and PATRIC databases [33, 34]. The testing for overrepresentation of certain functional categories among differentially expressed genes was performed using the GOseq package [76]. The results of RNA-seq and tables of raw reads were deposited in the GEO database under the accession number GSE66408.

### Quantitative real-time PCR

One microgram of total RNA was used for cDNA synthesis with random hexanucleotides and SuperScript III reverse transcriptase (Life Technologies). qPCR was performed using qPCRmix-HS SYBR (Evrogen, Russia) and the LightCycler 480 Real-Time PCR system (Roche, Switzerland); cycling conditions were as follows: 95° for 20s, 61° for 20s, 72° for 30s, repeat 40 times; primers are listed in Additional file 8: Table S8.

### Calculation of M-values

To calculate *M*-values, we chose 12 genes with medium and high expression levels in DM and DE cultures, and performed qRT-PCR on the RNA isolated from Log, DE, DM, and DL cultures using 16S rRNA as a reference gene (Additional file 4: Table S4). M-values for each of the genes were calculated according to equation:

$$ {M}_{Sample/ Log} = qPC{R}_{Sample/ Log}^{16S}\times \raisebox{1ex}{${\mathrm{RPKM}}_{Log}$}\!\left/ \!\raisebox{-1ex}{$RPK{M}_{Sample}$}\right. $$

Where *Sample* = DE, DM and DL. To estimate the change of mRNA content for each of the three comparisons, the average of *M*-values for 12 genes were calculated.

### Northern blotting analysis

For the detection of 23S rRNA, 2 μg of total RNA isolated from the Log and DM cultures was separated on a 1 % denaturing agarose gel in 1× MOPS buffer and transferred to Hybond N membranes (Amersham, UK) by transblotting. The membranes were hybridized overnight at 42 °C in ULTRAhyb-Oligo hybridization buffer (Life Technologies) with oligonucleotides NB5 and NB3, which were 5′-end radiolabeled (15 pmoles) using 10 μCi of [γ^32^P]-ATP and T4 polynucleotide kinase (Fermentas, Lithuania). After hybridization, the membranes were washed three times in 1× saline-sodium citrate buffer with 0.1 % SDS, and radioactivity was detected by exposure to an X-ray film overnight.

### Primer extension analysis

The oligonucleotide PE667 for mapping of the cleavage site on 23S rRNA was radiolabeled (10 pmoles) as described above. One microgram of total RNA isolated from the Log, DE, DM, and DL cultures was hybridized with 2 pmoles PE667 and reverse transcription was performed using SuperScript III reverse transcriptase (Life Technologies); the synthesized cDNA strands were separated on a 6 % denaturing polyacrylamide gel. To determine 23S rRNA cleavage site, the products of Sanger sequencing were run on four adjacent lanes. The sequencing of 23S rDNA was performed using the PE667 primer and Sequenase 2.0 DNA sequencing kit (Affymetrix, CA, USA), and radioactivity was detected by exposure to an X-ray film.

### Plasmid construction

Expression vectors were constructed by replacing the *Xba*I-*Hind*III fragment containing the *Hsp60* promoter in pMV261 [77] with the *Xba*I-*Hind*III fragment spanning −80 to −8 bp of the *rrnB* promoter from *M. smegmatis*. The promoter sequence was obtained by annealing oligonucleotides rrnB_F and rrnB_R (Additional file 8: Table S8). The vector was constructed so that small RNA could be inserted as a *Hind*III fragment downstream of the −10 region and the transcription would start at the mapped +1 nt with none or one additional nucleotide at the 5′ end. A synthetic transcriptional terminator was created by annealing oligonucleotides Term_F and Term_R to the insertion at the *Hind*III site downstream of sRNA 3′ end [35]. Small RNA was amplified by PCR using primers listed in Additional file 8: Table S8. Plasmids were transferred into mycobacteria by electroporation.

## Availability of supporting data

The data sets supporting the results of this article are available in the GEO data repository under the accession number GSE66408.

## Additional files

Additional file 1: Table S1. Mapping statistics for each of the sequenced samples. (XLSX 9 kb) Additional file 2: Table S2. RPKM values for each replicate in the corresponding cultures. (XLSX 797 kb) Additional file 3: Table S3. Correlation between samples. Spearman coefficients of correlation between each of the sequenced samples are shown. (XLSX 10 kb) Additional file 4: Table S4. Expression changes of selected genes and total mRNA transcriptome. (A) The expression of selected genes in the Log, DE, DM, and DL samples is presented as RPKM values (RNA-seq) and relative mRNA levels normalized to 16S rRNA (qPCR). (B) The expression of selected genes was determined by RNA-seq (RPKM values) and qPCR (relative to 16S rRNA) and presented as the ratio of the DE, DM, and DL values normalized to Log values. (C) Correlation between expression changes measured by RNA-seq and qRT-PCR. (D) Coefficient *M* representing the changes in the quantity of total mRNA relative to 16S rRNA. (XLSX 15 kb) Additional file 5: Table S5. Differential expression of protein-coding genes. The analysis of differential expression was performed with the edgeR software. Results for the following comparisons are shown: DE relative to Log, DM relative to DE, and DL relative to DM. Log2 fold change of gene expression, FDR-adjusted P-value for differential expression test, and *M*-value adjusted Log2 fold change are shown for every gene in each comparison. (XLSX 683 kb) Additional file 6: Table S6. Functional enrichments analysis. Functional enrichment analysis was performed using the GOseq package [76] and functional categories from the TubercuList and PATRIC databases [33, 34]. The lists of differentially expressed (up- and down-regulated) genes were generated by the comparison of the following samples: DE vs. Log, DM vs. DE, and DL vs. DM. For each functional category, the number of differentially expressed genes, the total number of genes in the category, and the adjusted p-value of overrepresentation are shown. (XLSX 38 kb) Additional file 7: Table S7. Candidate non-coding RNAs. Candidate non-coding transcripts were identified by visual analysis of the transcriptional profiles and designated according to the nomenclature suggested in [79]. For each candidate non-coding RNA its type, approximate coordinates, average RPKM value in each sample, original name, and references to the studies where the transcript has been described are presented. (XLSX 13 kb) Additional file 8: Table S8. Oligonucleotides used in the study. (XLSX 10 kb)
